# Initiating a network to support engagement between health researchers and schools: recommendations from an international meeting of schools engagement practitioners held in Kilifi, Kenya **

**DOI:** 10.12688/wellcomeopenres.15556.2

**Published:** 2020-07-07

**Authors:** Alun Davies, Grace Mwango, Bernard Appiah, James J. Callery, Vu Duy Thanh, Nozibusiso Gumede, Robert Inglis, Shane McCracken, Kestern Mkoola, Kagisho Montjane, Alice Ochanda, Charity Shonai, Kathryn Woods-Townsend

**Affiliations:** 1 Kenya Medical Research Institute (KEMRI) Wellcome Trust Research Institute, PO Box 230, Kilifi, 80108, Kenya; 2 Centre for Tropical Medicine and Global Health, Nuffield Department of Medicine, The University of Oxford, Oxford, UK; 3 Research Program on Public and International Engagement for Health, Texas A&M University School of Public Health, Texas, 77843, USA; 4 Centre for Science and Health Communication, PMB M71, Ministries, Accra, Ghana; 5 Mahidol Oxford Tropical Medicine Research Unit, Faculty of Tropical Medicine, Mahidol University, Bangkok, Thailand; 6 Oxford University Clinical Research Unit (OUCRU), 764 Vo Van Kiet, Quan 5, Ho Chi Minh City, Vietnam; 7 Science CEO Academy, Durban, KwaZulu Natal, South Africa; 8 Science Spaza, an initiative of Jive Media Africa, P.O.Box 22106, Mayor’s Walk, 3208, South Africa; 9 Gallomanor, 7-9 North Parade Buildings, Bath, BA1 1NS, UK; 10 Malawi-Liverpool-Wellcome Trust Clinical Research Programme, Queen Elizabeth Central Hospital, College of Medicine, P.O. Box 30096, Chichiri, Blantyre 3, Malawi; 11 Division of Human Genetics, Institute of Infectious Disease and Molecular Medicine, Faculty of Health Sciences, University of Cape Town, Cape Town, 7925, South Africa; 12 UNESCO Natural Sciences (SC/PCB), Regional Office for Eastern Africa, UN Gigiri Complex, Block C, Upper level, Nairobi, P.O. Box 30592, 00100, Kenya; 13 Zimbabwe Early Intervention in Psychosis, Plot P Arnott Road, Westgate, Harare, Zimbabwe; 14 Southampton Education School, Faculty of Social Sciences, University of Southampton, Southampton, UK; 15 NIHR Southampton Biomedical Research Centre, University of Southampton and University Hospital Southampton NHS Foundation Trust, Southampton, UK

**Keywords:** Community Engagement, Public Engagement, Health Research, Science, schools

## Abstract

Engagement between health researchers and local schools, or School Engagement, has become incorporated into the engagement strategies of many research institutions worldwide. Innovative initiatives have emerged within Wellcome Trust-funded African and Asian Programmes (APPs) and elsewhere, and continued funding from the Wellcome Trust and other funders is likely to catalyse further innovation. Engagement between *scientists* and schools is well-described in the scientific literature (1-4), however, engagement between *health researchers* and schools is much newer, particularly in sub-Saharan Africa, and rarely documented. In November 2018 the KEMRI-Wellcome Trust Research Programme (KWTRP) hosted an international workshop in Kilifi, Kenya, drawing on an emerging community of School Engagement practitioners towards exploring the broad range of goals for School Engagement, learning about the breadth of evaluation approaches and exploring the potential usefulness of establishing a practitioner network. The workshop was attended by 29 engagement researchers/practitioners representing 21 institutions from 10 countries in sub-Saharan Africa and South East Asia and the UK. Workshop sessions combining small group discussions with plenary presentations, enabled a range of goals, activities and evaluation approaches to be shared. This report summarises these discussions, and shares participant views on the possible functions of a network of School Engagement practitioners.

A breadth of ‘deep’ and ‘wide’ engagement activities were described addressing four broad goals: contributing to science education; capacity strengthening for health research; contributing to goals of community engagement; and health promotion. While wide approaches have greater outreach for raising student awareness, deeper approaches are more likely enable informed student views to be incorporated into research. All activities ultimately aimed at improving health, but also at supporting development in low- and middle-income countries through promoting science-career uptake. Participants identified a range of potential benefits which could emerge from a practitioner network: sharing experiences and resources; facilitating capacity strengthening; and fostering collaboration

## Background

Community engagement with health research is argued to be important to enhance the ethical conduct of health research ^5– 7^. Goals for engaging communities range from protecting communities from harms and exploitation, empowering autonomy for individual research decision-making, and enhancing the benefits of research participation to communities, to incorporating community views into research implementation ^8^. Previous discussions with community engagement practitioners have revealed a broader list of goals such as improving health care, contributing to community development, and raising awareness of research for recruitment ^9^. This broad range of goals have precipitated a range of engagement approaches in sub-Saharan Africa, from community bazaars to deliberative discussions and community advisory boards ^10^. More recently, health research institutions have explored engagement between researchers and local schools as a means of addressing some of the goals of community engagement ^11^.

The term ‘school engagement’ is frequently used to describe the degree of investment a student has in their school ^12^; however, in this article, ‘School Engagement’ with health research describes a range of activities which facilitates learning interactions between health researchers and primary and secondary school teachers and students. School Engagement approaches have included: attachment schemes and pre-university summer schools for students in Kenya and the USA ^11, 13, 14^; establishing long-term involvement of individual scientists with schools in Australia to enhance science lessons ^1, 15^; in-school education resources, coupled with school visits to institution laboratories to facilitate interactions between researchers and students in Kenya and the UK ^11, 16, 17^; and Young Persons’ Advisory Groups (YPAGs), which can potentially facilitate the incorporation of the unique perspectives of young people into research implementation in the UK and USA ^18– 20^. The goals of these approaches vary considerably from raising student awareness of health and research and gaining their insights into research, to stimulating an interest in science and research careers and demystifying the work of scientists.

The November 2018 international meeting for 29 School Engagement practitioners, held at the KWTRP, was the first meeting of its kind, bringing delegates together from 21 research institutions in 11 countries in South East Asia, sub Saharan Africa and the UK, where engagement activities between researchers and schools had been conducted. The workshop aimed broadly at sharing participant experiences of School Engagement, exploring implementation and evaluation approaches, and initiating a network of practitioners and academics, to strengthen practice in the field of School Engagement with health research. It was felt by the organizers (KWTRP School Engagement Programme (SEP)) that this would provide a foundation for articulating the contribution School Engagement makes to the goals of community/public engagement with health research. The specific objectives of the workshop were:

1. To share and map out the range of School Engagement with health research activities;
2. To map the range of goals for School Engagement with health research;
3. To share ethical dilemmas emerging from School Engagement and explore ways of addressing them;
4. To share experiences of School Engagement evaluation and to explore how other approaches such as the realist and theory of change approaches may be used for School Engagement evaluation; and
5. To explore potential functions and means of maintaining a network of School Engagement practitioners.

The purpose of this paper is to describe the range of School Engagement activities and evaluation approaches used by participants and their institutions, to map the range of goals for School Engagement from the participants’ perspective, and to explore practitioner perspectives on the potential usefulness of a School Engagement practitioner network and means of sustaining it.

### Workshop description

The workshop was funded through the Wellcome Trust’s Provision for Public Engagement. The Wellcome Trust is a major global health research funder with a funding portfolio of £4.3 billion in 2019, £56 million of which is invested in public engagement. The appetite for establishing a network and potential workshop discussion themes were explored prior to the Kilifi workshop with engagement practitioners from Malawi, South Africa, Kenya, Vietnam and Thailand at the Wellcome International Engagement meeting held in Vietnam in October 2018 and at the Global Health Bioethics Network regional meeting held in Malawi in September 2018. To elicit a diversity of perspectives on school engagement with health research, we invited a range of practitioners working in South East Asia, sub-Saharan Africa and the UK that we identified through Wellcome--funded partners and networks. We supplemented Wellcome Trust-funded practitioners with other delegates working if the field of school engagement identified through snowballing. Thus, our invitation list combined purposive and convenience approaches in an effort to represent diverse contexts and experiences. All participants and their affiliations are included in the author list and *Acknowledgement*.

This participatory collaborative school engagement workshop comprised a combination of presentations of School Engagement approaches and evaluation from all participating projects; video sharing sessions; learning sessions on applying theory of change to School Engagement; small group discussions and plenary reflections. Workshop discussion sessions were led by the main workshop facilitator (Alun Davies) and typically comprised the 5 small participants’ groups being given a 20-minute task for, for example, “brainstorm and list the goals of school engagement”. Small groups presented their summaries on flip charts to the larger plenary groups, who were able to reflect and share opinions on the group’s work. The data generated comprised group flipcharts and plenary flipchart summaries. This approach was used to share practitioner views, experiences and perceptions, in a similar way to a previous workshop, held in Kilifi, for community engagement and informed consent scholars ^9^.

***Mapping of engagement activities, and evaluation approaches***. The range of School Engagement activities and evaluation approaches were mapped through a combination of participant presentations, PowerPoint slides and follow-up summaries provided by email. The range of activities is shown in Table 1.

**Table 1.** Engagement between researchers and schools: approaches, goals and evaluation methods presented at the meeting.

Programme	Engagement approach(es)	Goals	Audience	Evaluation approach
Children Against Antimicrobial Resistance **Ghana**	• Story-telling • Using animated cartoons to facilitate learning • Picture drawing • Storytelling and picture drawing competition	• To raise awareness of anti-microbial resistance among school students • To nurture responsible use of antibiotics among students and their parents	375 students and their parents	Mixed methods evaluation: • Participant Observation • Interviews of purposively selected children and parents • Survey of randomly selected children and their parents
I’m a scientist, get me out of here! (IAS) UK, international.	• On-line dialogue between scientists (including health researchers) and students • Currently active in: Kenya, Vietnam, Spain, UK and Ireland	• Support Science Capital • Normalise scientists • Raise awareness of science research • Raise an appreciation of science	20,000 students annually	Mixed methods evaluation: Surveys Focus Group Discussions (FGDs) Interviews Content analysis
KEMRI-Wellcome Trust School Engagement Programme **– Kenya**	• School Leavers’ 3-month attachment Scheme (9 students per year) • Student laboratory visits • Scientists visiting schools - career talks • On-line engagement ( IAS) • Primary science clubs • Young Persons’ Advisory Groups • Annual school science competitions	• Raising an interest in science and science related careers • Raising student awareness of locally conducted research • Nurturing a respect for the community among researchers • Drawing on student perspectives to feed into research implementation	4000 + students annually	Mixed methods evaluation: • Pre/post student surveys (with control group) • Qualitative interviews and FGDs with teachers, researchers, students and parents • Participatory video and participant observation – students make their own videos to share their experiences of the attachment
LifeLab, Southampton **UK**	Module of work, includes: • Teacher CPD • Lessons based on research from local university/hospital (local population based), looking at how scientists do their work (coming up with ideas, planning experiments, collecting data, analysing data and writing/presenting results) • Day visit to LifeLab with hands on practical activities and a ‘Meet the Scientist’ sessions • Health qualification (20 students), accredited by national body (Royal Society of Public Health) and related to national qualifications (GCSE level) • Early LifeLab – modules of work including teacher CPD and then flight cases of resources to go out to primary schools	• Changing health behaviours • Equipping young people with the knowledge, understanding and skills to know why lifestyle choices are important for current and future health and to be empowered and resilient to enable different lifestyle choices • Equipping teachers with the confidence to have ‘healthy conversations’ with students and their parents to support health behaviour change • Raising an interest in science and science related careers • Raising student awareness of locally conducted research	2500 students annually	Outcome measures collected via: • Student surveys including RCTs: ◦ Knowledge, attitudes behaviour in relation to science/health ◦ Self-efficacy/motivation/confidence in making healthy diet choices/physical activity choices ◦ Interest in science/scientists • Food Frequency questionnaires • Height/weight measurements • Physical activity trackers • Pre/post surveys to explore engagement impact on researchers • Qualitative methods • FGDs and interviews with students, teachers and parents • Numbers of Health champions trained • Health campaigns in school
Malawi Liverpool Wellcome Trust (MLW) - **Malawi**	• School Leavers attachment programme • Permanent public exhibition • Outreach exhibition • Science for all project • School science clubs	• Generating a community of engaged young people who are health champions in their school (and local communities) (agents of change?)	1,600 students	• Video diaries with students • Pre/post self-administered student questionnaires • Short reports from students • FGDs and Informal discussions with teachers & students • Career trackers for students
Mahidol Oxford Tropical Research Unit (MORU), **Thailand**	• Cambodia – Village drama about malaria and prevention/elimination methods performed by school children ^22– 25^	• Educate communities about malaria & childhood vaccination • Promote traditional & ethnic arts • Introduce a new MORU research station at Siam Pang Health Centre (2018)	1,900 participants, 35,000 attendees (adults & children), across 45 villages.	Mixed methods evaluation: • In depth interviews with community members, community leaders and performers. • Attendance numbers • Participant observation • Log of activities and challenges
mGEN Africa **Zimbabwe**	• On-line mobile app facilitating engagement between genomics researchers and school students • Face-to-face engagement: open days; teacher workshops; job-shadows; and school competitions	• Learn about science careers • Learn about genomics research • Enable students to share their views about research	400 students	• Pre and Post surveys.
OUCRU- **Vietnam**	• Science clubs (hands-on science activities; scientific projects and science debates). • Science magazine page (weekly science articles and monthly Ask the scientists) • Online annual science competition • IAS, online engagement • Science visits (school students visit OUCRU and OUCRU researchers visit schools)	• Developing scientific capacity and an appreciation for science and research through experiencing science activities and interacting with researchers. • Building capacity of Change Makers (CM) (researchers, teachers and university students) to promote science and research in schools • Influencing policies that support wider and sustainable opportunities for school students to participate in science and research	30,000 students	• Pre and Post surveys. • In-depth interviews, focus group discussions with school students, teachers, and researchers. • Google analysis. • Participatory activities: ranking, drawing.
UNESCO- Girls STEM project **Kenya**	• Mentorship talks on STEM careers • Facilitate industry visits • Teacher sensitisation workshops	• Demystify science and unlock the hidden potential in girls for enhanced participation in STEM fields while at the same time making them agents of change in their respective communities.	2000 students from 167 schools 170 teachers	• Monitoring and Evaluation includes impact assessment through performance and STEM uptake tracking of mentored students
Science CEO Academy South Africa	• Learner support and motivational talks • Career guidance • Girls in STEM programme • E-learning • Workshops	• To raise student awareness of science related careers and attract students to further education in Science Technology Engineering and Maths (STEM • Strengthen maths, science and technology teacher capacity • Attract more girls into STEM careers • Assist learners to excel in STEM related subjects at Secondary School level.	6000+ students since inception	• Pre and Post academic surveys • In-depth interviews with principals and teachers • Interviews with learners • Comparison on academic performance of the schools in the programme and those not in a programme. • Tracking of learner’s tertiary qualification choices post-secondary school.
Science Spaza and Hip-hop health, **South Africa**	• Science clubs – peer-to-peer learning • Science comics and videos • Creating Hip-hop music with youth to stimulate dialogue and learning	• To grow appreciation for the power of scientific research to transform lives among those with little or no lived experience of science culture • Stimulate dialogue about water-borne disease	Science clubs in 140 schools	• Post engagement discussions • Content analysis of created poems/songs
Z-Factor Drama competitions Zimbabwe	• School Drama competitions • Quizzes	• Creating a platform whereby the community can freely discuss issues with regards to mental health.	2 community wards	• Activity reports and attendance registers • Audio/video recordings • Judge scoresheets • Focused group discussions • Key informant interviews • Scaled pre and post questionnaires • Indepth individual interviews

IAS, I’m A Scientist, Get Me Out of Here – online engagement platform; CPD, continued professional development; FGD, focus group discussion; RCT, randomised control trial; GCSE, general certificate in secondary education (UK); OUCRU, Oxford University Clinical Research Unit; UNESCO STEM, The United Nations Educational, Scientific and Cultural Organization – Science Technology Engineering and Mathematics.

**Figure 1.**
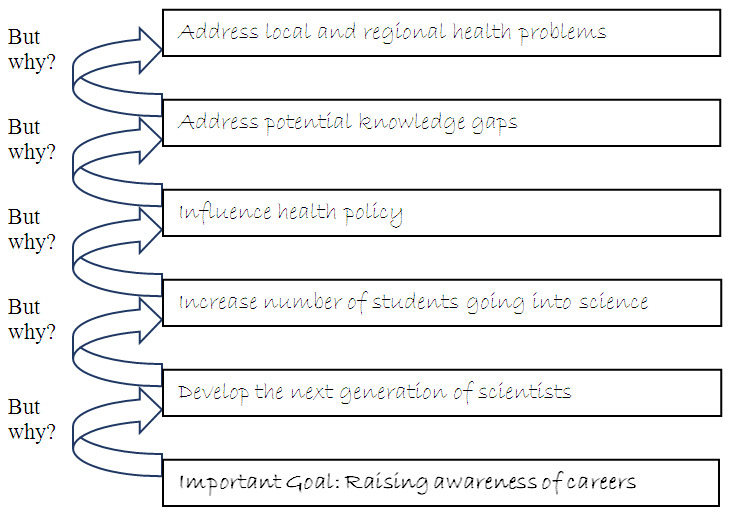
Tool for interrogating goals (example drawn from the workshop).

***Mapping the goals of School Engagement***. In small groups, workshop participants were tasked with brainstorming goals for engagement between health researchers and schools based on their experience. These goals were shared in the plenary and group reflection sessions then captured and added to the list of resulting goals. Following this activity, the small groups were asked to select their most important goals and further interrogate the purpose of these goals using a modified approach to the ‘five whys’ technique ^21^, using the tool illustrated in Figure 1.

**Figure 2.**
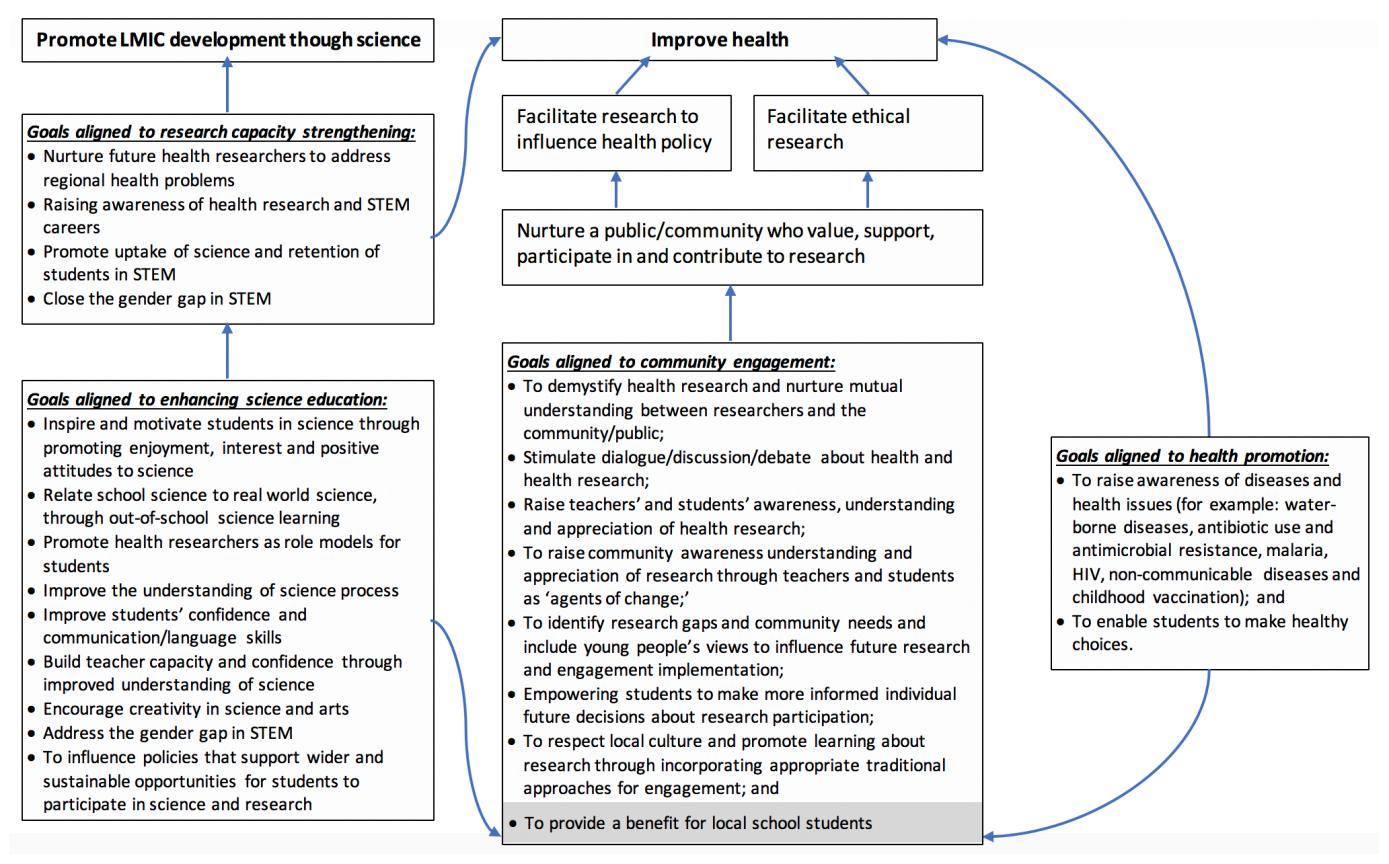
Goal map for School Engagement activities generated from the practitioner workshop.

In the example shown in Figure 1, though group members initially struggled with the sequence from the 2 ^nd^ to the 3 ^rd^ goals up, in plenary they clarified that engagement activities were aimed at attracting and developing a cadre of researchers, who would in the future be able to conduct research which would contribute to health policy and ultimately improve health. The purpose of this exercise was to explore ‘higher level’ or overarching goals for engaging schools with health research. In this exercise, participants placed their individual goals in the lower box, and asked themselves, “why do we want to achieve this goal?” The goal yielded from this question, arrived through group discussion and consensus, was subsequently written into the 1 ^st^ box up. This process was repeated up to four further times, or until a range of ‘higher level goals’ were achieved across the groups. Reflections about these higher-level goals were shared in plenary, and emerging patterns of overarching goals school engagement goals were seen across all groups. These emerging higher-level goals formed the basic structure for a goal map ( Figure 2). Drawing from each groups’ ‘5-whys’ template, the general goals of school engagement were able to be grouped as subsidiaries to the overarching goals. Further reflections on the resulting goal map were shared among the group through email and the goal map ( Figure 2) was amended accordingly.

***Mapping participant views about an School Engagement practitioner network***. Similar to the initial brainstorming of engagement goals, in order to gather participant views on what they could share and draw from a School Engagement network, participants in small groups were asked to discuss the following questions:

- Why do we need a School Engagement network?
- What skills and resources would you like to draw on and share in the network?
- How can we share materials and resources?

Group responses summarised on flipcharts were shared in plenary allowing all participants to reflect further on the potential utility of a School Engagement network.

***Capturing participant experiences and views***. All views and reflections were captured on flipcharts and through note-taking. The notes and flipchart data were transcribed, summarized and consolidated post workshop. Participants were given an opportunity to reflect on the summaries through email.

## Workshop findings

The findings are presented in four areas: School Engagement activities; evaluation approaches; the goals of School Engagement; and the value of a network of practitioners.

***School Engagement activities***. Participants described a range of engagement activities facilitated mainly in sub-Saharan Africa and south East Asia, but also in other parts of the world including the UK, ranging from in-depth engagement with a few school students over a prolonged period of time, to short one-day visits for larger groups, to large-scale engagement through popular media and the internet. These activities are summarized below and described in more detail in Table 1.

- Student visits to laboratories and exhibitions visits for interactive/practical activities and discussions with scientists;
- Fully resourced modules of work, linked to the school science curriculum, taught in school and supported by a hands-on practical activity day at separate purpose-built educational facility;
- Follow-up school sessions and lesson suggestions with related support materials;
- Scientists visiting schools – to give health, career and motivational talks to students;
- On-line engagement on a platform called “I’m a scientist, get me out of here” where students can ask questions and chat to scientists through text over two-week events;
- Primary science clubs where schools are supported to establish in-school science clubs supported by scientist visits and support material;
- YPAGs – which involves regular meetings with a group of 10–20 young people in order to facilitate discussion into practical and ethical aspects of research;
- Science competitions – this can involve a large number of schools in on-line or face-to-face interactions such as quizzes;
- Regular features in popular (national) magazines for children;
- Teacher trainings and workshops – to support good science-teaching practice;
- A health qualification (related to national UK GCSE level qualifications);
- Using comics, videos, hip-hop music and storytelling to stimulate dialogue and learning; and
- Creating and performing village based dramas to stimulate discussions about health.

The way in which these engagement activities were rolled out also ranged from single short-term under 2-year projects involving less than five schools, to large longer-term, larger school programmes comprising multi-engagement approaches established over 5–10 years. Clearly, these diverse approaches to School Engagement had a correspondingly broad range of goals.

***Evaluation approaches***. Participants described a wide range of evaluation approaches and methods, ranging from cluster-randomized controlled trials ^26^ to mixed method evaluations combining quasi-experimental designs with qualitative and participatory approaches, such as participatory video ^27, 28^. With a few exceptions, most projects reported that they had not published their findings in peer reviewed journals, but instead used evaluation to report back to funders and to contribute to learning about the weaknesses and strengths of the projects. This would inform future development of the activities/programmes. The full range of evaluation approaches and methods is described in Table 1.

***The goals of engagement between researchers and schools***. These goals of engagement between researchers and schools shared during the workshop can be grouped into four broad overlapping and interrelated categories: community engagement goals; science education goals; research/science capacity strengthening goals; and health promotion goals. Each of these goal categories were articulated as contributing ultimately to two overlying aims: facilitating development in LMICs through encouraging the uptake of science careers among young people; and facilitating ethical research to improve health. The pathways through which the categories of goals contribute to the main overlying aims can be represented as a goal map illustrated in Figure 2.

***Goals aligned to community engagement with research***. Some of the goals in this category resonate with goals described for community engagement with health research described elsewhere ^9^. Goals which aligned to the broader goals of engagement were described in three ways: firstly as contributing an ‘operational goal’ of helping to facilitate research to influence health policy, through fostering an informed and supportive public; secondly, to address ‘intrinsic’ engagement goals, such as respecting local culture and creating fora where members of the public/community could express their views about research; and thirdly to address ethical principles of research, through supporting individual informed consent through a better understanding of research, and providing benefits to students/schools in the form of enhancing science education and promoting health. For many workshop participants, engaging school students as members of the community/public was deemed important in its own right but was also described as important in terms of the students’ potential to become ‘agents of change’ influencing wider understanding of and attitudes towards health research among their families, friends and wider communities.

Creating opportunities for discussion, dialogue and debate were described as means of facilitating the students’ learning about research, but also in some cases, for example through YPAGs, to facilitate the learning in the other direction: to feed student perspectives into research implementation and nurture researchers’ understanding of public/community perspectives.

The underlying purpose of all of these goals aligned to community engagement goals were primarily articulated as a means of nurturing a public/community who value, support, participate in and contribute to research. Nurturing a community/public and support for research was described as a contributor to facilitating research to influence health policy, which would in turn, contribute to better health

***Goals aligned to enhancing science education***. That health research draws on fundamental scientific methods and principles, highlights an opportunity for school engagement practitioners to take advantage of the overlap between community engagement and science education goals. Raising student awareness of how clinical trials work, for example, presents an opportunity to strengthen school science curricula content on ‘fair testing’, control groups, and how vaccines work.

Educational goals of interactions between scientists and schools have been widely described in science education literature elsewhere (see for example Braund and Reiss ^29^) and similar goals were shared for engagement between health researchers and schools. These goals included: improving students’ understanding of scientific processes (improving scientific literacy); offering opportunities for students to relate school science to ‘real-world science’; promoting positive attitudes to science; de-mystifying scientists; and strengthening science teachers’ capacity and confidence through offering opportunities to improve their understanding of science.

For most workshop participants, School Engagement was described as a means of drawing from research institutions’ scientific or arts backgrounds and resources, both human and material, to contribute to enhancing school students’ science education experiences. Through contributing to local education, research institutions could provide a benefit to local communities/publics, which in itself can be also be thought of as addressing a goal of Community Engagement. This benefit, widely described as a being a valid and important output of engagement in itself, was also described by some workshop participants as a means of nurturing a community supportive of research implementation. This highlights overlaps between the categories of goals.

***Goals aligned to research capacity strengthening***. Providing opportunities for students to interact with researchers, as well as contributing to addressing science education goals, was viewed as a means of raising students’ interest and aspirations for pursuing science and research-related careers. Though overlapping with science education goals, these goals were deemed important contributing factors in themselves for attracting talented young people to science/research-related careers and therefore potentially important in strengthening capacity for future research. Strengthening science/research capacity was primarily described as a means of ultimately improving health, but also as a means of promoting development through science specifically in LMICs.

***Health promotion goals***. Underlying health promotion goals was a notion that health research institutions, because of their obvious expertise in local prevalent disease and health, were well placed to support health promotion initiatives such as: empowering young people to make healthier choices to reduce their risk of developing chronic diseases (such as heart disease and diabetes) later in life; raising awareness of sexually transmitted infections including HIV, and strategies to prevent infections; minimizing the risk of water-borne diseases through good hygiene; malaria prevention and childhood vaccination; and antimicrobial resistance. Again clear overlaps between the goals of science education and health promotion enable practitioners to kill two birds with one stone, for example, raising STI or water-borne disease prevention awareness can address both curricular and health promotion goals simultaneously. In a similar way to enhancing science education, health promotion was described in terms of both benefiting individual students and in terms of improving community/public health.

***Networking***. Participant feedback for the workshop evaluation highlighted that learning from others about the range of goals and engagement approaches, and sharing practical and ethical challenges faced during implementation felt very enriching and helpful. As summarised in Table 2, group discussions, subsequent plenary presentations and workshop evaluation feedback yielded four broad categories of motivations for establishing a network of School Engagement practitioners: to draw on network expertise for skill-sharing and capacity strengthening; to facilitate discussions to share experiences; to facilitate collaboration and support for fundraising; and to share resources. These categories and their associated objective raised during the meeting are listed below. Overwhelmingly, delegates felt that regular workshop meetings would provide the best opportunities for achieving goals such as capacity and skills development, sharing experiences, problem solving and brainstorming. Many delegates felt that though several other networks presented opportunities for sharing experiences, the growing diversity of School Engagement approaches merited its own network meeting.

**Table 2.** Goals and activities of School Engagement?

Overall goals	Specific goals and activities
To draw on network expertise for skill- sharing and capacity strengthening for school engagement:	• Sharing engagement approaches/practice – to learn about new approaches/best practice; • Sharing evaluation/research methodologies – quantitative, qualitative and participatory methods to strengthen the evidence and argument for School Engagement; • Provide opportunities for practitioners to develop presentation and science communication skills; • To offer opportunities to learning about important skills such as stakeholder engagement; and • To create a database/directory of different skills within the School Engagement practitioner group.
To share experiences and facilitate discussions on School Engagement:	• To nurture mutual-encouragement through offering participants an opportunity to have their work appreciated; • To provide a platform to share successes, failures and lessons learned (to avoid duplication of mistakes); • To provide a platform to share share, discuss and offer guidance on ethical and practical challenges emerging in School Engagement activities; and • To provide opportunities to brainstorm, share and develop new engagement activities.
To facilitate collaboration and support for proposal writing and fundraising	• To draw on credible advisors/consultants/experts for funding applications from the pool of experts within the network; • To better understand the funding landscape and strengthen funding applications and collaborative awards; • Collaboration towards generating new cross-programme School Engagement ideas; and • To grow the network – identify new partners/collaborators, and opportunities.
To share resources	• Evaluation tools and documents; • Published literature and meeting presentations; • Training resources (e.g. teacher and researcher); and • Guidance/advice for capacity strengthening and trainings.

Table content summarized from workshop discussion and plenary reflection.

Though delegates felt that face-to-face workshops could not be replaced, several other modes of communication were suggested to supplement this. The online Mesh network was felt to be potentially useful for sharing resources such as theories of change, evaluation documents and activity curricula. It was also felt that group and one-to-one communication could be facilitated through Skype, email, and social media.

## Discussion

We acknowledge that this is by no means an exhaustive description of all projects facilitating School Engagement with health research. However, it provides a snapshot of some of the diverse activities conducted in parts of sub-Saharan Africa, South East Asia and the UK. This broad range of activities has diverse goals which can be categorised into four main overlapping groups: research-related community engagement goals; goals aligned to enhancing science education; goals aligned to research and science capacity strengthening; and health promotion goals. More fundamentally, in LMICs all these goals aim at nurturing a country’s development through supporting science and facilitating ethical research to improve health.

**Figure 3.**
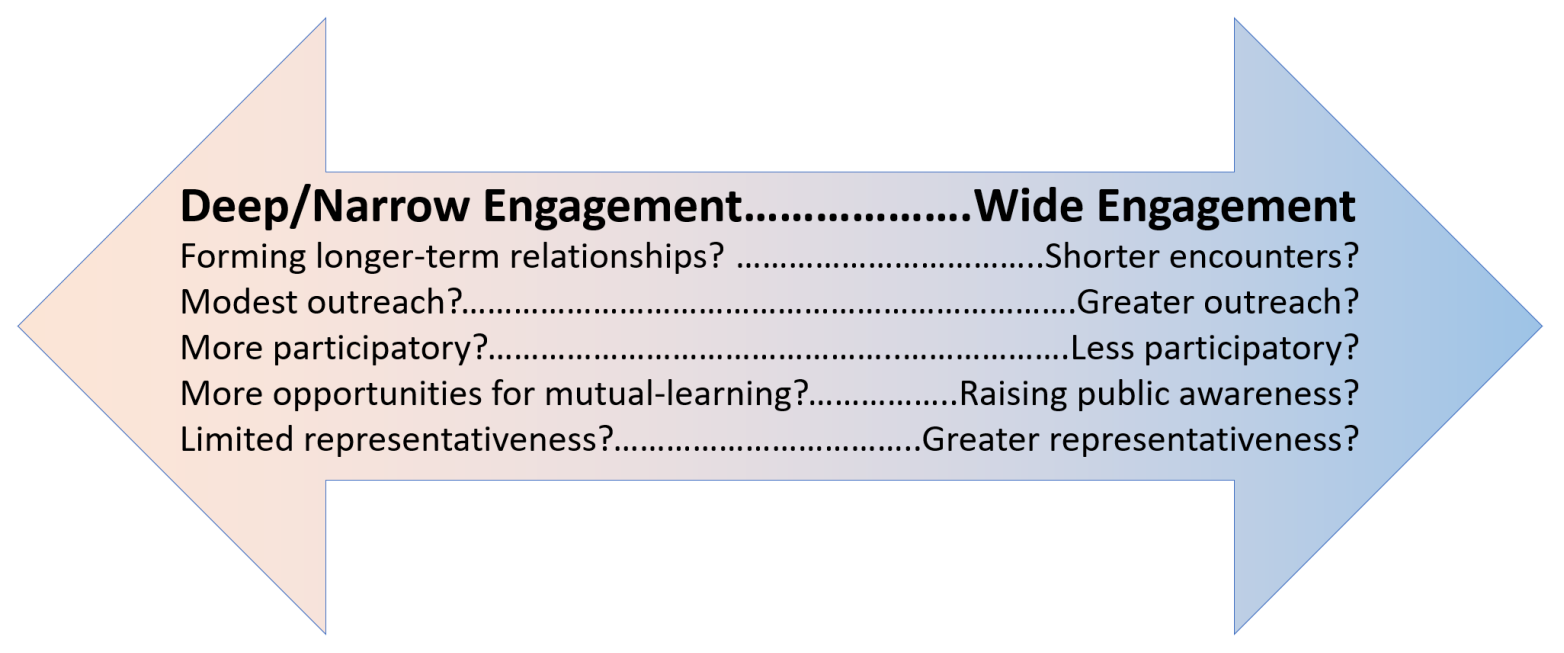
Continuum of deep to wide engagement.

As well as ranging in terms of approaches and goals, engagement activities also varied tremendously in terms of project ‘depth’ and ‘width’. Holliman and Davies ^30^ point to the importance of considering the ‘depth’ of engagement, in terms of relative ratios of students to participating researchers and duration of engagement activity, as a means of “ *moving beyond the seductive siren of reach*”. Comparing a range of engagement activities between universities and schools, they argue that the deeper the engagement per student, the more ‘ *meaningful and direct*’ the interactions will be, but at greater resource costs per student (including researcher time). At our workshop, while ‘deep’ activities such as YPAGs ^19^ and attachment schemes ^11^, involved prolonged engagement over an extended period of time with low student to researcher ratios, wider activities such as one-off lab-tours for considerably larger student groups (see for example Davies, Mbete *et al.* ^27^) and the OUCRU Viet Nam National Science magazine initiative (see Table 1), involved much larger numbers (the latter 40,000 students) for considerably shorter interactions. Some activities described in this article could be argued to be both wide and deep simultaneously. For example, the LifeLab project, through a combination of teacher professional development training, student activity days at a hospital facility, and 10 follow-up classroom-based lessons ^16^, could be described as both deep and wide interaction. This illustrates that school engagement activities lie along a continuum of deep to wide engagement. Figure 3 presents an argument that along the continuum from ‘deep’ to ‘wide’ school engagement, longer-term relationships are developed with smaller numbers of students, offering a greater scope for participatory approaches and mutual learning, but at the cost of a smaller outreach, narrower representation and limited opportunities for participatory work.

Because of the few UK-based School Engagement activities that were presented at the workshop, any contextual differences between UK- and LMICs-based engagement approaches and prioritisation of the goals concerning school engagement cannot be ascertained from this paper and thus need further exploration.

Given that there is a need, particularly in LMICs, to attempt to balance meaningful ‘deep’ engagement, with the need to distribute potential school engagement benefits to the largest possible number of schools, we feel that both ‘deep’ and ‘wide’ forms of engagement are equally important, but arguably serve different purposes. For example, whilst a deeper engagement through regular YPAG meetings, can potentially facilitate the incorporation of unique youth perspectives into research implementation in contexts where their voices are taken seriously by research leads ^20^, there is growing evidence of the benefits of larger-scale short institutional visits to students knowledge, attitudes and aspirations ^27, 28, 31, 32^.

During workshop discussions, a great emphasis was placed on the importance of engagement being of ‘benefit’ to students and schools, and of identifying and minimising any potential inconveniences or disruptions. Regarding benefits, research conducted in LMICs has the capacity of generating collateral benefits for research participants and communities hosting research ^33^. While individual research participants may acquire direct ‘collateral benefits’ such as access to medicine or healthcare, which may not usually be available ^34^, communities may also benefit as a result of research institutions’ presence in communities through for example, improved healthcare, access to health education materials or the provision clean water and boreholes ^35, 36^. From the individual researcher or research institution’s perspective, contributing to local education can be seen as a collateral benefit. This resonates with previous findings from Kenya where researchers felt that motivating school students in science provided an appropriate means of ‘giving back’ to local communities in return for their careers in science being supported by local communities ^27^. Whilst the importance of engagement being beneficial for students in terms of, for example, inspiring positive attitudes towards science or promoting healthy behaviours cannot be understated, engagement practitioners were concerned that any such benefits must outweigh potential disruption to children and school curriculum delivery. Other emerging challenges, particularly in LMIC contexts, include: the potential for engagement to raise school teachers’ expectations which are challenging to meet by researchers (e.g. building/equipping school laboratories); limitations to a research institutions capacity for school outreach may leave uninvited schools feeling neglected; and limited school enrolment leading to out-of-school youth gaining no benefits from engagement ^11, 28^. Further research is needed to document any unintended perverse outcomes and to strengthen the evidence that engagement is beneficial to students in terms of some of the overarching goals of School Engagement listed in this article: enhancing science education; promoting health behaviour; and upstream science capacity strengthening. Further, though researchers, teachers and students may perceive School Engagement overall as beneficial, it is essential that there is a continued engagement with representatives of broader communities on their views about School Engagement: what are the most valued approaches; and how does School Engagement weigh up against other potential forms of collateral community benefit?

Whilst community engagement with health research has limitations in terms of its capacity to address all community needs ^9^, we argue that School Engagement can make a substantial contribution to addressing goals of community engagement, capacity strengthening and health promotion, while at the same time benefitting host communities through supporting local education. The workshop has illustrated the value of networking across a range of projects to highlight similarities and differences between projects and to understand the nature and range of engagement approaches. Participants at this initial School Engagement meeting were mainly restricted to engagement practitioners attached to research institutions, educationalists and science communicators. Whilst questions about future network participants remain, for example: a sole focus on engagement between health researchers and schools or a broader outlook on engagement with science; or, should the network extend to science teachers and children? participant thirst for sharing, learning and collaborating highlighted the value of further developing this practitioner group.

## Conclusion

The workshop identified four main School Engagement goals and several School Engagement approaches and evaluations. Participants identified the value of a School Engagement practitioner’s network to share resources, lessons and experiences. We argue that given the potential benefits of School Engagement, there is a need to strengthen its practice and research across LMICs.

## Data availability

No data are associated with this article.
