# Supplementary material for: Initiating a network to support engagement between health researchers and schools: recommendations from an international meeting of schools engagement practitioners held in Kilifi, Kenya
Source: Wellcome Open Res. 2020 Jul 7;4:180. Originally published 2019 Nov 18. [Version 2] doi: 10.12688/wellcomeopenres.15556.2 (PMC7372533; doi:10.12688/wellcomeopenres.15556.2)
Supplement: Peer review history

# Peer review: Reviewer response for version 2

**DOI:** 10.21956/wellcomeopenres.17674.r39447

**Published:** 2020-07-20

**Authors:** Ann Grand

**Affiliations:** 1 The Open University, Milton Keynes, UK

Does the article adequately reference differing views and opinions?

Partly

Are all factual statements correct, and are statements and arguments made adequately supported by citations?

Yes

Is the Open Letter written in accessible language?

Yes

Where applicable, are recommendations and next steps explained clearly for others to follow?

Partly

Is the rationale for the Open Letter provided in sufficient detail?

Partly

Reviewer Expertise:

Engaged research, public engagement with research, qualitative methods, facilitation, science communication.

I confirm that I have read this submission and believe that I have an appropriate level of expertise to confirm that it is of an acceptable scientific standard.

# Peer review: Reviewer response for version 1

**DOI:** 10.21956/wellcomeopenres.17036.r37811

**Published:** 2020-02-17

**Authors:** Ann Grand

**Affiliations:** 1 The Open University, Milton Keynes, UK

This is an interesting article demonstrating the sharing of experience and expertise among a wide range of groups involved in engagement work with schools around health research. It is particularly valuable in reflecting engagement in countries and communities beyond the ‘usual suspects’ of the UK, USA and Europe. As the authors note, much of the learning from such engagement activities is used in reports to funders, rather than also underpinning research papers, so it is useful and interesting to see it here.

 However, if this work is to be truly useful in supporting further work in other countries, I would suggest there needs to be more detail in the methodology. For example, there’s nothing wrong with using a convenience sample of Wellcome Trust-funded researchers but it would be useful to know how the participants were identified and brought together. The authors have listed the combination of activities used at the workshop, but there is no detail on how they operated – were they facilitated or organic, how were the activities initiated, who decided on the questions to be addressed. Likewise, more detail on how the evidence was captured; for example who captured the discussion on flipcharts – participants or facilitators. We also don’t know how the data were analysed and summarised.

 There is a degree of pre-judging in some of the aims of the workshop, particularly the notion of a practitioner network; at the beginning of page 4, there is an aim to ‘initiate a network of practitioners’, which suggests it is foreordained, whereas in the list of objectives, one is to examine the utility of such a network. Throughout the article, engagement is posited as a benefit to communities and I believe the article would have been stronger with the inclusion of a discussion of the challenges and limitations of engagement, which I am sure the participants considered.

 The figures are helpful in illustrating potential tools for mapping and discussing goals but both need more context. The actual example given in Figure 1 is useful but there is no logical link between goals 3 and 4, and I feel sure that in reality this would have been discussed and clarified. In Figure 2, I am left wondering how the division between the two goals emerged.

 The discussion on page 11 doesn’t always match the boxes in Figure 2. For example, Figure 2 has a box labelled ‘goals aligned to community engagement’, whereas the discussion has ‘research-related community engagement goals’, which is not quite the same thing. In this section, the important point that the categories of goals overlap is rather lost in the middle; all the categories overlapped, so I suggest this could have been accorded more clarity.

 The tensions and questions around the competing claims of depth and width in engagement form a sizeable section of the Discussion but are only brought in in this section. Given the wide range of activities summarised in Table 1, this dichotomy could have formed a useful discussion point for the participants, which would make its presence in the Discussion more relevant.

 I would like to have seen more evidence from the data gathered during the workshop (the authors say there are no data but the flipcharts, recordings, etc. are all data sources). Hearing the authentic voices of the participants would greatly strengthen the conclusions and recommendations. However, there is no mention of ethical approval from any institution, so perhaps this is why no data are offered.

Does the article adequately reference differing views and opinions?

Partly

Are all factual statements correct, and are statements and arguments made adequately supported by citations?

Yes

Is the Open Letter written in accessible language?

Yes

Where applicable, are recommendations and next steps explained clearly for others to follow?

Partly

Is the rationale for the Open Letter provided in sufficient detail?

Partly

Reviewer Expertise:

Engaged research, public engagement with research, qualitative methods, facilitation, science communication.

I confirm that I have read this submission and believe that I have an appropriate level of expertise to confirm that it is of an acceptable scientific standard, however I have significant reservations, as outlined above.

# Response

**Published:** 2020-06-24

**Authors:** Alun Davies

**Affiliations:** KEMRI-Wellcome Trust Research Programme, Kenya

Many thanks to **Ann Grand** from The Open University, Milton Keynes, UK for taking time to read and review our manuscript. On behalf of the authors I would like to thank you for your valuable contributions. Our responses are included underlined and italicized below.

1)Throughout the article, engagement is posited as a benefit to communities and I believe the article would have been stronger with the inclusion of a discussion of the challenges and limitations of engagement, which I am sure the participants considered. *A paragraph has been included in the discussion to describe emerging challenges of school engagement referenced in the literature.*

2) There needs to be more detail in the methodology.

- How the participants were identified and brought together? E.g. there’s nothing wrong with using a convenience sample of Wellcome Trust-funded researchers but it would be useful to know. *A combination of convenience and purposive approaches were used to identify workshop invitees. This has been clarified in the methods.*
- The authors have listed the combination of activities used at the workshop, but there is no detail on how they operated – were they facilitated or organic, how were the activities initiated, who decided on the questions to be addressed. *The questions addressed at the workshop were brainstormed beforehand with engagement practitioners at the Global Health Bioethics network Africa Regional and Wellcome International meetings in 2018. Pre-planned sessions were led by a facilitator.  Both these points have been clarified in the methods section*
- More detail on how the evidence was captured; for example, who captured the discussion on flip-charts – participants or facilitators. We also don’t know how the data were analysed and summarized. *Evidence was captured through group flip-chart presentations and summaries and notes were taken during plenary discussions. This has been addressed and described in more detail in the methods section.*
- There is a degree of pre-judging in some of the aims of the workshop, particularly the notion of a practitioner network; at the beginning of page 4, there is an aim to ‘initiate a network of practitioners’, which suggests it is foreordained, whereas in the list of objectives, one is to examine the utility of such a network. *It’s true to some extent that the appetite for such a network was explored prior to the Kilifi workshop with engagement practitioners at the Global Health Bioethics network Africa Regional and Wellcome International meetings in 2018.  In the workshop we wanted to explore the potential usefulness of such a network and how it would operate and have clarified this in the text. We have re-worded the workshop objectives accordingly.*

3) The figures are helpful in illustrating potential tools for mapping and discussing goals, but both need more context. The actual example given in Figure 1 is useful but there is no logical link between goals 3 and 4, and I feel sure that in reality this would have been discussed and clarified. *The small workshop group who produced the ‘5-whys’ example in figure 1 described their sequence in plenary and clarified* *how combined goals 2 and 3 (creating a future generation of scientists and increasing the number of students taking up science) would translate to strengthening the capacity for future researcher,  who would in turn be able to conduct research to influence health policy. This context has been added to the figure. This is admittedly long-winded, but essentially highlights how concrete activity goals are related to more abstract aspirational ‘higher-level’ goals.*

4)In Figure 2, I am left wondering how the division between the two goals emerged. *In the amended draft article, we have described how the 5-whys method and plenary reflections on these enabled ‘higher-level goals’ to be derived. Post workshop, individual goals were grouped into these higher-level goal categories and affirmed with delegates through email. This has been clarified in the amended article. All authors felt that these goal categories were a useful way of classifying more specific goals*

5) The discussion on page 11 doesn’t always match the boxes in Figure 2. For example, Figure 2 has a box labelled ‘goals aligned to community engagement’, whereas the discussion has ‘research-related community engagement goals’, which is not quite the same thing. In this section, the important point that the categories of goals overlap is rather lost in the middle; all the categories overlapped, so I suggest this could have been accorded more clarity. *This comment has been very useful in making us articulate the overlaps more carefully. Overlaps have been highlighted in two sections and the title in the discussion has been changed to “Goals aligned to community engagement with research”*

6) The tensions and questions around the competing claims of depth and width in engagement form a sizable section of the Discussion but are only brought in in this section. Given the wide range of activities summarized in Table 1, this dichotomy could have formed a useful discussion point for the participants, which would make its presence in the Discussion more relevant. *In retrospect, we agree that though ‘depth’ and ‘width’ were apparent in delegate presentations and discussed briefly in plenary, it would have been a good addition to the themes of the workshop for further discussion. In the article we show that this continuum was formed drawing on the referenced article by Holliman and Davies and through collating data for table 1 from all PowerPoint presentations and emails following the meeting. In response to Piotrowski et al, we have added a diagram to clarify the continuum.*

7) I would like to have seen more evidence from the data gathered during the workshop (the authors say there are no data but the flip-charts, recordings, etc. are all data sources). Hearing the authentic voices of the participants would greatly strengthen the conclusions and recommendations. However, there is no mention of ethical approval from any institution, so perhaps this is why no data are offered. *At the outset, as opposed to a formal study guided by a research protocol, consent forms etc., this initiative was conceived primarily as a workshop. During the initial workshop session, it was suggested that the discussion would make a useful contribution to the engagement literature, and there was strong agreement among all delegates. Discussions were not recorded, but group discussion summaries were developed, and participants confirmed they were comfortable with these summaries being drawn upon in writing the paper.  All workshop participants (authors and acknowledged participants) saw a draft of the paper (shared by email) and were happy with its’ submission.  As such, adding verbatim quotes is unfortunately not possible.*

# Peer review: Reviewer response for version 1

**DOI:** 10.21956/wellcomeopenres.17036.r37473

**Published:** 2020-01-30

**Authors:** Sally Theobald, Helen Piotrowski, Shahreen Chowdhury

**Affiliations:** 1 Liverpool School of Tropical Medicine, Liverpool, UK; 2 Department of International Public Health, Liverpool School of Tropical Medicine (LSTM), Liverpool, UK

This is an engaging and accessible article clearly showing learning from a diverse range of practitioners on processes, goals and learning from school engagement activities with health research, across a range of contexts from sub-Saharan Africa, East Asia and the UK. It is an interesting and important read given the growing interest in this area and the relative lack of published literature on this; as stated in the article, most of those involved in school engagement activities had not published their work, but rather used evaluations to inform reports to funders.

 The abstract is detailed on the workshop, methods and participants and covers the four emerging broad goals for schools engagement including contributing to science education; capacity strengthening for health research; contributing to goals of community engagement; and health promotion. However, there is less detail on the learning and key points in the discussion (e.g. trade offs between deep and wide approaches and on the depth of engagement) which we felt would be useful to include in the abstract too.

 In the background it would be useful to know more about where work on school engagement has taken place, for example, which contexts and where does the literature cited come from? Throughout the paper, there are some inconsistencies in the language used in describing where the work has taken place; for example, the authors refer to work in “Africa” but it would be good to share where in Africa, such as in the references in the background. However, the term sub-Saharan Africa is used in the discussion, which provides more specificity, and Table 1 clearly details where in Africa the work has been done.

 It may also be useful to comment on whether there were any differences between the different contexts in prioritisation of the goals concerning school engagement. For example, in Table 1, it seems there was more engagement with art, such as cartoons, story-telling and theatre, in LMICS compared to the UK contexts; while in South Africa and Zimbabwe, there was more of a focus on gender in encouraging girls into STEM careers. Table 1 is a comprehensive summary of engagement approaches, goals, audiences, methods for evaluation and demonstrates the wide range of initiatives and perspectives that were included in the workshop. This is a very useful resource for others who may be interested in taking forward school engagement with health research.

 Figure 1 and 2 are interesting and illustrate the tools used to map the goals. However, it would be good to expand on the discussions and dilemmas pertaining to these and whether there are any links between the two figures. If so, the title of Figure 2 could be more descriptive if this summarises the themes generated from the workshop with the network of practitioners. The tension and importance of both deep and wide approaches to school engagement are interesting in the discussion and we wondered whether these could also be displayed visually?

 Throughout the Open Letter, school engagement is positioned as a benefit to individuals, communities and researchers or institutions. Whilst the authors note that stakeholders highlighted benefits of school engagement activities should outweigh disruptions to children and curriculum, there was little discussion presented about any of the challenges of school engagement activities, especially who is engaged and who is potentially missed. However, the Open Letter helps facilitate a discussion on how school engagement activities can support wider community engagement strategies and highlights the appetite amongst practitioners to support mechanisms which will enable sustained shared learning within this very important field. There was also an interesting focus on students becoming agents of change and the role they can play in community engagement. It is clear from this letter that establishing a network in school engagement can help facilitate shared learning; now it is important to have a dialogue about how this could be sustained and what stakeholders could contribute. A collaboration such as this could have some important and innovative outputs.

Does the article adequately reference differing views and opinions?

Partly

Are all factual statements correct, and are statements and arguments made adequately supported by citations?

Yes

Is the Open Letter written in accessible language?

Yes

Where applicable, are recommendations and next steps explained clearly for others to follow?

Yes

Is the rationale for the Open Letter provided in sufficient detail?

Yes

Reviewer Expertise:

We are 3 reviewers Sally Theobald, Helen Piotrowski, Shahreen Chowdhury with expertise in health systems strengthening, community engagement and equity.

We confirm that we have read this submission and believe that we have an appropriate level of expertise to confirm that it is of an acceptable scientific standard.

# Response

**Published:** 2020-06-24

**Authors:** Alun Davies

**Affiliations:** KEMRI-Wellcome Trust Research Programme, Kenya

Many thanks to **Helen Piotrowski** **, **Shahreen Chowdhury****and ****Sally Theobald**** from the Liverpool School of Tropical Medicine (LSTM), Liverpool, UK, for taking time to read and review our manuscript. On behalf of the authors I would like to thank you for your valuable contributions. Responses to your individual comments/suggestions have been shown in italics and underlined below:

1. The abstract is detailed on the workshop, methods and participants and covers the four emerging broad goals for schools engagement including contributing to science education; capacity strengthening for health research; contributing to goals of community engagement; and health promotion. However, there is less detail on the learning and key points in the discussion (e.g. trade offs between deep and wide approaches and on the depth of engagement) which we felt would be useful to include in the abstract too. *We have added a sentence in the abstract to address this.*
2. In the background it would be useful to know more about where work on school engagement has taken place, for example, which contexts and where does the literature cited come from? *The countries where school engagement activities are referenced have been included in the background.*
3. Consistency Throughout “Africa” - sub-Saharan Africa is used in the discussion, which provides more specificity, and Table 1 clearly details where in Africa the work has been done. *We have clarified sub-Saharan Africa and South East Asia throughout the article.*
4. It would be useful to comment on whether there were any differences between the different contexts in prioritization of the goals concerning school engagement. - Table 1, it seems there was more engagement with art/cartoons/story-telling/ theatre, in LMICs compared to the UK contexts. *Representation of engagement projects in the UK at the meeting was not sufficient to come to such a conclusion, and this has been clarified in the article.*
5. Figure 1 and 2 are interesting and illustrate the tools used to map the goals. However, it would be good to expand on the discussions and dilemmas pertaining to these and whether there are any links between the two figures. If so, the title of Figure 2 could be more descriptive if this summarizes the themes generated from the workshop with the network of practitioners. *In the amended draft article, we have described how the 5-whys method and plenary reflections on these enabled ‘higher-level goals’ to be derived. Post workshop, individual goals were grouped into these higher-level goal categories and affirmed with delegates through email. This has been clarified in the amended article. All authors felt that these goal categories were a useful way of classifying more specific goals. The title of Figure 2 has been amended to read: “* *Goal map for School Engagement activities* *generated from the practitioner workshop”*
6. The tension and importance of both deep and wide approaches to school engagement are interesting in the discussion and we wondered whether these could also be displayed visually? *Thanks for this suggestion – we have presented a visual representation of the continuum between wide and deep engagement.*
7. Throughout the Open Letter, school engagement is positioned as a benefit to individuals, communities and researchers or institutions. Whilst the authors note that stakeholders highlighted benefits of school engagement activities should outweigh disruptions to children and curriculum, there was little discussion presented about any of the challenges of school engagement activities, especially who is engaged and who is potentially missed. *A paragraph has been included in the discussion to describe referenced emerging challenges of school engagement.*
8. It is clear from this letter that establishing a network in school engagement can help facilitate shared learning; now it is important to have a dialogue about how this could be sustained and what stakeholders could contribute. A collaboration such as this could have some important and innovative outputs. *Thanks for this comment, we agree.*
